# On the yield criterion of porous materials by the homogenization approach and Steigmann–Ogden surface model

**DOI:** 10.1038/s41598-023-38050-8

**Published:** 2023-07-06

**Authors:** Chenyi Zheng, Hongzhen Wang, Yali Jiang, Gaohui Li

**Affiliations:** Huadong Engineering Corporation Limited, Hangzhou, 311122 Zhejiang China

**Keywords:** Materials science, Mathematics and computing, Nanoscience and technology

## Abstract

In this work, we investigate the yield criterion of nanoporous materials by using homogenization approach and Steigmann–Ogden surface model. The representative volume element is proposed as an infinite matrix containing a tiny nanovoid. The matrix is incompressible, rigid-perfectly plastic, von Mises materials and nanovoids are dilute and equal in size. First, the constitutive of microscopic stress and microscopic strain rate is established based on the flow criterion. Secondly, according to the Hill’s lemma, the relationship between the macroscopic equivalent modulus and the microscopic equivalent modulus is established by homogenization approach. Thirdly, the macroscopic equivalent modulus containing the Steigmann–Ogden surface model including surface parameters, porosity and nanovoid radius is derived from the trial microscopic velocity field. Finally, an implicit macroscopic yield criterion for nanoporous materials is developed. For surface modulus, nanovoids radius and porosity studies are developed through extensive numerical experiments. The research results in this paper have reference significance for the design and manufacture of nanoporous materials.

## Introduction

Nanoporous materials have outstanding material properties, including high porosity^1^, large specific surface area, high thermal conductivity, high electrical conductivity, high energy adsorption and corrosion resistance. Due to the superior properties of nanoporous materials, related research articles have also been developed, including the study of effective modulus^2,3^, elastic response^4–7^ and strength analysis of nanoporous materials^8,9^.

Among these studies, most of the literature is limited to the effect of surface and interface mechanical responses on elastic properties, while lack of focus on strength criteria for nanoporous materials, which has important implications for the design and fabrication of nanoporous materials. In terms of the yield criterion of porous materials, Gurson^1^ proposed the famous Gurson yield criterion based on the trial microscopic velocity field from the perspective of energy. The effect of void ratio on the macroscopic yield criterion is fully considered in the Gurson yield criterion, so that the macroscopic yield criterion depends on both the macroscopic equivalent stress and the macroscopic average stress. Since the effects of void interactions and coalescence were ignored, Tvergaard^10^ improved the Gurson yield criterion by calibrating using finite element unit cell calculations. Tvergaard and Needleman^11^ further extended the macroscopic yield criterion according to a set of elastic-plastic constitutive relations, known as the famous GTN model.

For the research on the yield criterion of nanoporous materials, scholars mainly carry out two methods: numerical and theoretical^12,13^. As an important numerical method, finite element theory is also used in the study of the yield criterion of nanoporous materials. Nasir et al.^14^ combined a Gurson-type yield function including void size effects with finite element theory to predict the forming limit of aluminum materials based on the interfacial stress of the membrane around spherical voids. The results show that a smaller void size leads to an increase in the ductility limit of the material. Espeseth et al.^15^ presented a numerical study of a finite element-based unit cell consisting of a single spherical void embedded in a matrix material, with size effects represented by a porous plastic model with voids. Espeseth investigated the effect of the intrinsic length scale of the matrix material on void growth and coalescence under a range of stress states. Unlike classical finite element theory, Usman et al.^16^ investigated the effect of void shape on the micromechanisms of void growth by using discrete dislocation plasticity simulations and using the extended finite element method (XFEM) to model displacement discontinuities.

In the theoretical study of nanoporous materials, a typical method is to couple the strain gradient theory on the Gurson model. By combining strain gradient theory and the classical Gurson model, Li et al.^17^ proposed a macroscopic yield criterion for spherical representative volume elements (RVEs) for axisymmetric tensile traction. Monchiet et al.^18^ extends Gurson’s yield criterion based on strain gradient theory and derives an approximate closed-form macroscopic yield function. On the basis of the strain gradient theory, the research on the yield criterion of nanoporous materials under complex working conditions is further developed. Niordson and Tvergaard^19^ recently generalized the theory of strain gradient plasticity with dissipative gradient effects to finite strain in order to quantify the size-scale effect on void growth under different loading conditions. Ban^20^ considers the influence of deformation damage on the basis of strain gradient plasticity theory and proposed a modified incremental constitutive model to characterize the coupling effect of size and damage in micro-metallic materials.

In addition to strain gradient theory, coupling surface theory at the interface of representative volume element (RVE) is also a recent popular practice. Based on the traditional Gurson model, Dormieux and Kondo^21^ coupled the Gurtin-Murdoch surface model at the inner surface of the spherical void to deduce the macroscopic yield function of nanoporous materials and explored the effect of surface parameters on the macroscopic yield loci. Monchiet^22^ used the same method to study the macroscopic yield function of nanoporous materials with viscoplastic matrix. Next, Monchiet and Kondo^23^ further studied the yield criterion of nanoporous materials under ellipsoidal RVE. However, the Gurtin-Murdoch surface model only considers the surface tensile stress, while ignoring the existence of the surface compressive stress^24,25^. In order to supplement this deficiency, Zheng and Mi^13^ obtained the macroscopic yield criterion of nanoporous materials based on the Steigmann–Ogden surface model and explored the mechanism of surface bending moment.

Different from the Gurson model, the homogenization approach establishes the macroscopic yield function of nanoporous materials from the perspective of energy, which depends on the relationship between the macroscopic equivalent modulus and the matrix modulus. Due to the existence of critical points of elasticity and plasticity, scholars can derive the macroscopic yield function of nanoporous materials from the perspectives of elastic limit and plastic flow. Zhang et al.^26^ derived the macroscopic yield function of nanoporous materials considering the Gurtin-Murdoch surface model from the perspective of elastic limit and studied the influence of surface elastic parameters on the macroscopic yield function. Chen^27^ used the same method to study the macroscopic yield function of nanoporous materials with columnar RVE. Zheng and Mi^28^ combined the homogenization theory and the Gurson model to derive the macroscopic yield function of multi-scale nanoporous materials.

Besides the analysis of elastic limit, Dormieux and Kondo^8^ firstly derived the macroscopic yield function of nanoporous materials from the perspective of plastic flow, in which the imperfect interface is replaced by a thin film. In order to solve the problem of plastic modulus, Brach et al.^29^ based on the layered method solved the equivalent plastic modulus under different layers of matrix and deduced the macroscopic yield criterion of nanoporous materials, respectively. Subsequently,^30^ further studied the macroscopic yield function of nanoporous materials with general matrix under axisymmetric conditions. However, the above analysis ignores the influence of surface compressive stress on the imperfect interface.

The purpose of this paper is to continue the previous work, consider the influence of surface compressive stress on imperfect interface from the perspective of plastic flow and obtain the macroscopic yield criterion of nanoporous materials. Firstly, based on the plastic flow criterion, the plastic constitutive of the von Mises matrix is studied. Secondly, through the homogenization approach, the equivalent shear modulus of the matrix is obtained. Thirdly, according to the law of conservation of energy, the relationship between the macroscopic equivalent modulus and the microscopic equivalent modulus is derived. Finally, the macroscopic yield criterion of the nanoporous material considering the Steigmann–Ogden surface model is obtained through the trial velocity field.

The remainder of this paper is structured as follows. Section 2 details the homogenization approach and the derivation of the macroscopic equivalent modulus containing Steigmann–Ogden surface model. According to the Hill’s lemma, the macroscopic yield criterion of nanoporous materials is obtained. In Section 3, extensive parametric studies are conducted in order to examine the effects of nanovoids surface bulk modulus, shear modulus, bending rigidity, nanovoids radius and porosity on the yield loci of nanoporous materials. In Section 4, concluding remarks are made.

## Method of solution

**Figure 1:**
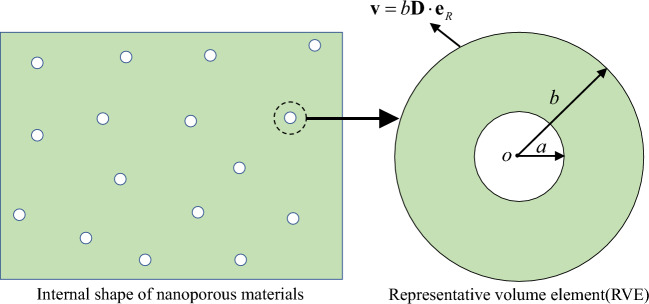
The nanoporous materials containing spherical nanovoids and the representative volume element (RVE).

Figure 1 shows the nanoporous materials containing multiple nanovoids inside. All multiple nanovoids are assumed to share the same radius and be far apart but to distribute randomly in space. To intercept a nanovoid as representative volume element (RVE), a standard Mori-Tanaka model will be considered, which is completely consistent with the image on the right side of Figure 1. *a* and *b* are denoted as the inner and outer radii of the RVE, respectively. Among them, the magnitude of the outer radius is much larger than the inner radius ($a \gg b$) . The volumes occupied by the void, matrix and RVE are denoted by $V_1$, $V_2$ and $V_3$, respectively. The outer boundary of the RVE is subjected to an arbitrary axisymmetric macroscopic strain rate ($\textbf{D}$).

### Homogenization approach

Let us consider that the matrix of the RVE satisfies the von Mises yield criterion. The yield surface of the matrix is denoted by $g \left( {{\sigma }} \right)$:

1 $$\begin{aligned} g \left( {{\sigma }} \right) = \frac{3}{2}{{{\sigma }}_d}:{{{\sigma }}_d} - \sigma _Y^2 \le 0, \end{aligned}$$

where ${{\sigma }}_d$ and $\sigma _Y$ stand for the microscopic deviatoric stress and microscopic yield stress for the whole RVE. By means of the plastic flow criterion, the microscopic strain rate can be easily obtained:

2 $$\begin{aligned} {\textbf{d}} = {\dot{\lambda }} \frac{{\partial f\left( {{\sigma }} \right) }}{{\partial {{\sigma }}}} = 3{\dot{\lambda }} {{{\sigma }}_d}, \end{aligned}$$

where ${\dot{\lambda }}$ denotes plastic flow rate. For the convenience of subsequent derivation, the fourth-order mean projection tensor ${\mathbb {J}}$ and the deviatoric projection tensor ${\mathbb {K}}$ can be introduced here. Their tensor form can be represented by index notation:

3a-c $$\begin{aligned} {J_{ijkl}} = \frac{1}{3}{\delta _{ij}}{\delta _{kl}},\quad {K_{ijkl}} = {I_{ijkl}} - {J_{ijkl}},\quad {I_{ijkl}} = \frac{1}{2}\left( {{\delta _{ik}}{\delta _{jl}} + {\delta _{il}}{\delta _{jk}}} \right) , \end{aligned}$$

where $\delta _{ij}$ and $I_{ijkl}$ indicate identity second-order tensor and identity fourth-order tensor. Using the projection tensor, Equation (2) can be easily simplified to

4 $$\begin{aligned} \mathbf{{d}} = 3{\dot{\lambda }} {\mathbb {K}}:{{\sigma }}. \end{aligned}$$

Simultaneously multiplying the deviatoric projection tensor on both sides of the Equation (4):

5 $$\begin{aligned} {\mathbb {K}}:\mathbf{{d}} = 3{\dot{\lambda }} {\mathbb {K}}:{\mathbb {K}}:\mathbf{{\sigma }}. \end{aligned}$$

It is not difficult to obtain the expression of the microscopic deviatoric stress by the fourth-order deviatoric projection tensor:

6 $$\begin{aligned} {{{\sigma }}_d} = {\mathbb {K}}:{{\sigma }} = \frac{1}{{3{\dot{\lambda }} }}{\mathbb {K}}:\mathbf{{d}}. \end{aligned}$$

Substituting Eq. (6) into the yield function (1) of the matrix in limit state, the plastic flow rate (${\dot{\lambda }}$) can be derived

7 $$\begin{aligned} {\dot{\lambda }} = \frac{1}{{{\sigma _Y}}}\sqrt{\frac{1}{6}{} \mathbf{{d'}}:\mathbf{{d'}}}, \end{aligned}$$

where $\mathbf {d'}$ denotes the microscopic deviatoric strain rate. Knowing the form of microscopic stress and microscopic strain rate, the maximum plastic dissipation at any point in the RVE matrix can be expressed as

8 $$\begin{aligned} \pi \left( \mathbf{{d}} \right) = {{\sigma }}:\mathbf{{d}} = {\sigma _Y}{d_{eq}}. \end{aligned}$$

Here, we exploit the principle of plastic dissipation consistency to solve for the equivalent strain rate ($d_{eq}$) at the limit state

9 $$\begin{aligned} {d_{eq}} = \sqrt{\frac{2}{3}{} \mathbf{{d'}}:\mathbf{{d'}}}. \end{aligned}$$

The plastic constitutive equation of the microscopic stress can be derived by the plastic flow criterion

10 $$\begin{aligned} {{\sigma }} = \frac{{\partial \pi \left( \mathbf{{d}} \right) }}{{\partial \mathbf{{d}}}} = \frac{2}{3}\frac{{{\sigma _Y}}}{{{d_{eq}}}}{} \mathbf{{d'}}, \end{aligned}$$

where $\pi \left( \mathbf{{d}} \right)$ denotes the yield function of the matrix in terms of microscopic strain rate with the Equations (6, 7). Here we introduce the fourth-order plastic constitutive tensor (${\mathbb {C}}_2$) of the matrix to support the following derivation

11 $$\begin{aligned} {{\sigma }} = {{\mathbb {C}}_2}:\mathbf{{d}} = 2{\mu _2}^\prime \left( {\mathbf{{d'}}} \right) {\mathbb {K}}:\mathbf{{d}}. \end{aligned}$$

Since the matrix is incompressible, the plastic constitutive of the microscopic stress can be simplified by the fourth-order deviatorial strain projection tensor. The shear modulus of plasticity ${\mu _2}^\prime \left( {\mathbf{{d'}}} \right)$ is a function of the microscopic deviatoric strain rate. By Equations (10, 11), it is not difficult to obtain the functional expression of the plastic shear modulus

12 $$\begin{aligned} 2{\mu _2}^\prime \left( {\mathbf{{d'}}} \right) = \frac{2}{3}\frac{{{\sigma _Y}}}{{{d_{eq}}\left( {\mathbf{{d'}}} \right) }}. \end{aligned}$$

The non-constant nature of the plastic shear modulus can greatly increase the difficulty of solving the dissipation. To solve this puzzle,^8^ proposed microscopic equivalent modulus shear modulus by homogenization approach

13 $$\begin{aligned} 2{\mu _2} = \frac{2}{3}\frac{{{\sigma _Y}}}{{{{\tilde{d}}}_{eq}}}. \end{aligned}$$

where $\mu _2$ is the average plastic shear modulus. We introduce a reference deviatoric strain rate, defined as

14 $$\begin{aligned} {{{{\tilde{d}}}}_{eq}} = \sqrt{\frac{2}{3}\overline{\mathbf{{d'}}:\mathbf{{d'}}} }. \end{aligned}$$

The expression of the average operator is

15 $$\begin{aligned} {\bar{\cdot }} = \frac{1}{V}\int _V { \cdot dV}. \end{aligned}$$

The Hill’s lemma is presented

16 $$\begin{aligned} \mathbf{{{{\Sigma }}:D}} = \left( {1 - f} \right) \overline{\mathbf{{{{\sigma }}:d}}}, \end{aligned}$$

where the average operator here acts on the macroscopic representative volume of nanoporous materials, as shown in the left figure of Figure 1.

The plastic dissipation of the RVE from the macro perspective is expressed as

17 $$\begin{aligned} {\Pi \left( \mathbf{{D}} \right) } = {{\Sigma }}:\mathbf{{D}} = \mathbf{{D}}:\left( {3{\kappa _{3 }}{\mathbb {J}} + 2{\mu _{3 }}{\mathbb {K}}} \right) :\mathbf{{D}}={{\Sigma }}:\left( {\frac{1}{{3{\kappa _3}}}{\mathbb {J}} + \frac{1}{{2{\mu _3}}}{\mathbb {K}}} \right) :{{\Sigma }}. \end{aligned}$$

For macroscopic RVE, the equivalent bulk and shear plastic moduli are assumed to be constants and denoted as $\kappa _3$ and $\lambda _3$. Since the outer boundary of RVE is subjected to a uniform strain rate, the macroscopic strain rate is constant.

Combined with equations (13,14), the plastic dissipation of the RVE from a micro perspective is expressed as

18 $$\begin{aligned} \overline{\pi \left( \mathbf{{d}} \right) } = \overline{\mathbf{{{{\sigma }}:d}}} = \overline{2{\mu _2}{} \mathbf{{d:}}{\mathbb {K}}{} \mathbf{{:d}}} = 2{\mu _2}\overline{\mathbf{{d':d'}}} = \frac{1}{3}\frac{{\sigma _Y^2}}{{{\mu _2}}}. \end{aligned}$$

Combined with the Hill’s lemma and energy perturbation (17,18), the relationship between the macroscopic strain rate and the microscopic strain rate at the minimum potential energy is established

19 $$\begin{aligned} {{\Sigma }}:\left( {\frac{1}{{3\delta {\kappa _3}}}{\mathbb {J}} + \frac{1}{{2\delta {\mu _3}}}{\mathbb {K}}} \right) :{{\Sigma }} = \left( {1 - f} \right) \frac{{\sigma _Y^2}}{{3\delta {\mu _2}}}. \end{aligned}$$

The macroscopic yield criterion can be further established

20 $$\begin{aligned} 3\frac{{\mu _2^2}}{{\kappa _3^2}}\frac{{\delta {\kappa _3}}}{{\delta {\mu _2}}}{\left( {\frac{{{\Sigma _m}}}{{{\sigma _Y}}}} \right) ^2} + \frac{{\mu _2^2}}{{\mu _3^2}}\frac{{\delta {\mu _3}}}{{\delta {\mu _2}}}{\left( {\frac{{{\Sigma _{eq}}}}{{{\sigma _Y}}}} \right) ^2} - 1 + f = 0, \end{aligned}$$

where

21a-c $$\begin{aligned} {\Sigma _m} = \frac{{\mathrm{{tr}}\left( {{\Sigma }} \right) }}{3},\quad {\Sigma _{eq}} = \sqrt{\frac{3}{2}{{\Sigma '}}:{{\Sigma '}}} ,\quad {{\Sigma '}} = {{\Sigma }} - {\Sigma _m}{} \textbf{I}. \end{aligned}$$

From equation (20), it can be observed that the macroscopic yield criterion depends only on the porosity (*f*), the microscopic equivalent shear modulus ($\mu _2$) and the macroscopic equivalent bulk and shear moduli ($\kappa _3$ and $\mu _3$).

### Macroscopic equivalent bulk modulus ($\kappa _3$)

For any macroscopic strain rate, the strain rate field in the main direction can be obtained by coordinate transformation. As a basic form, only the axisymmetric case is considered in this paper. The macroscopic strain rate of the Cartesian coordinate system established in the principal direction is expressed as:

22 $$\begin{aligned} \mathbf{{D}} = \left[ {\begin{array}{*{20}{c}} {{D_m} + {D_e}}&{}\quad 0&{}\quad 0\\ 0&{}\quad {{D_m} + {D_e}}&{}\quad 0\\ 0&{}\quad 0&{}\quad {{D_m} - 2{D_e}} \end{array}} \right] , \end{aligned}$$

where the macroscopic mean strain rate and the macroscopic deviatoric strain rate are denoted by $D_m$ and $D_e$, respectively. Among them, the macroscopic equivalent bulk and shear moduli are generated only by the macroscopic mean strain rate and the macroscopic deviatoric strain rate, respectively.

Now, we will establish that the microscopic velocity field is generated only by the macroscopic mean strain rate:

23a,b $$\begin{aligned} v_R^1 = {F_1}R + {G_1}\frac{{{a^3}}}{{{R^2}}},\quad v_\varphi ^1 = v_\theta ^1 = 0, \end{aligned}$$

23c,d $$\begin{aligned} v_R^2 = {F_2}R + {G_2}\frac{{{a^3}}}{{{R^2}}},\quad v_\varphi ^2 = v_\theta ^2 = 0, \end{aligned}$$

where the superscripts 1 and 2 denote the inclusions and matrix of the microscopic RVE, respectively. The microscopic strain rate can be obtained by geometric equations

24a,b $$\begin{aligned} d _{ij}^1 = \frac{1}{2}\left( {v_{i,j}^1 + v_{j,i}^1} \right) ,\quad d _{ij}^2 = \frac{1}{2}\left( {v_{i,j}^2 + v_{j,i}^2}. \right) \end{aligned}$$

Establish the constitutive equation of microscopic stress

25a,b $$\begin{aligned} \sigma _{ij}^1 = {\lambda _1}d _{kk}^1{\delta _{ij}} + 2{\mu _1}d _{ij}^1,\quad \sigma _{ij}^2 = {\lambda _2}\varepsilon _{kk}^2{\delta _{ij}} + 2{\mu _2}d _{ij}^2, \end{aligned}$$

where $$\lambda _{1,2} = {{\left( {3\kappa _{1,2} - 2\mu _{1,2} } \right) } /3}$$.

There are four unknown coefficients on the microscopic velocity field that need to be determined by boundary conditions. The kinematic equations should be satisfied on the outer boundary.

26 $$\begin{aligned} v_R^2\left| {_{R = b}} \right. = {D_m}b \end{aligned}$$

Considering that the velocity field at the center of the sphere cannot be singular, the form of the unknown coefficient $G_1$ should be

27 $$\begin{aligned} {G_1} = 0. \end{aligned}$$

At the interface, the continuity condition of the velocity field should be guaranteed

28 $$\begin{aligned} v_R^1\left| {_{R = a} = } \right. v_R^2\left| {_{R = a}}. \right. \end{aligned}$$

Steigmann-Ogden governing equations^5^ for the force balance condition across a solid interface is described as

29 $$\begin{aligned} {[}{{\sigma }}{]} \cdot \mathbf{{n}} = {\nabla _S} \cdot {\varvec{\tau }} + {\nabla _S} \cdot \left( {\left( {{\nabla _S} \cdot \mathbf{{M}}} \right) \mathbf{{n}}} \right) - \left( {{\nabla _S} \cdot \mathbf{{n}}} \right) \mathbf{{n}} \cdot \left( {{\nabla _S} \cdot \mathbf{{M}}} \right) \mathbf{{n}}, \end{aligned}$$

where $\nabla _S$, $\varvec{\tau }$, $\textbf{M}$ and $\textbf{n}$ denote the surface projection gradient in spherical coordinates, surface stress, surface bending moment and unit outer normal vector of the sphere, respectively. For the convenience of solving, the second-order surface projection tensor $T_{ij}$ and normal projection tensor $N_{ij}$ are introduced

30a,b $$\begin{aligned} {T_{ij}} = {\delta _{ij}} - {n_i}{n_j}, \quad {N_{ij}} = {n_i}{n_j}. \end{aligned}$$

It is easy to find that the surface projection tensor and the normal projection tensor are orthogonal and normal.

31a-c $$\begin{aligned} {T_{ij}}{T_{jk}} = {T_{ik}},\quad {T_{ij}}{N_{jk}} = 0,\quad {N_{ij}}{N_{jk}} = {N_{ik}}. \end{aligned}$$

Split gradient operator into normal direction and surface direction

32 $$\begin{aligned} \frac{\partial }{{\partial {x_j}}} = {N_{ij}}\frac{\partial }{{\partial {x_i}}} + {T_{ij}}\frac{\partial }{{\partial {x_i}}}, \end{aligned}$$

where surface gradient operator $\nabla _S$ is written as ${T_{ij}}\frac{\partial }{{\partial {x_i}}}$ in index notation. The surface bending moment is expressed as

33a-c $$\begin{aligned} {M_{ij}} = {\zeta _s}{\kappa _{kk}}{T_{ij}} + 2{\chi _s}{\kappa _{ij}},\quad {\kappa _{ij}} = - \frac{1}{2}\left( {{\vartheta _{k,u}} + {\vartheta _{u,k}}} \right) {T_{iu}}{T_{kj}},\quad {\vartheta _i} = {T_{ij}}{n_k}{v_{k,j}}. \end{aligned}$$

The surface stress is expressed as

34a-b $$\begin{aligned} {\tau _{ij}} = {\lambda _0}d_{kk}^s{\delta _{ij}} + 2{\mu _0}d_{ij}^s,\quad d_{ij}^s = {T_{ik}}{d_{kl}}{T_{lj}}. \end{aligned}$$

Through the above efforts, the interface stress condition (29) with surface effect can be rewritten as

35 $$\begin{aligned} {{[} \sigma ]_{ij}}{n_i} = {\tau _{ij,i}} + {T_{lu}}{\left( {{M_{kl,k}}{n_j}} \right) _{,u}} - {T_{ik}}{n_{i,k}}{n_l}{M_{ml,m}}{n_j} \end{aligned}$$

By calculation, it can be obtained

36 $$\begin{aligned} {T_{ik}}{n_{i,k}} = \frac{2}{a}. \end{aligned}$$

The surface stress condition of the Steigmann-Ogden model can be further rewritten as

37 $$\begin{aligned} {{[} \sigma ]_{ij}}{n_i} = {\tau _{ij,i}} + {T_{lu}}{\left( {{M_{kl,k}}{n_j}} \right) _{,u}} - \frac{2}{a}{M_{ml,m}}{n_j}{n_l}. \end{aligned}$$

Using the trial velocity field, the slope change vector $\vartheta _i$ is written as

38 $$\begin{aligned} {\vartheta _i} = {T_{ij}}{n_k}{v_{k,j}} = 0. \end{aligned}$$

From this, it can be judged that the surface bending moment does not participate under the action of the macroscopic mean strain rate due to $M_{ij}=0$. Therefore, the surface stress condition of Steigmann-Ogden surface model will degenerate into the surface stress condition of Gurtin-Murdoch surface model.

39 $$\begin{aligned} {[} \sigma {]}_{ij}{n_{i}} = {\tau _{ij,i}} \end{aligned}$$

Through the above four boundary conditions (26, 27, 28, 39), the four unknown coefficients ($F_1,F_2,G_1,G_2$) contained in the microscopic velocity field can be uniquely determined.

Define the average microscopic strain rate as^2^:

40 $$\begin{aligned} {{\bar{d}} _{ij}} = \frac{1}{{2V}}\int _S {\left( {{n_i}{v_j} + {n_j}{v_i}} \right) } dS. \end{aligned}$$

The average strain rate of the matrix and the average strain rate of the inclusions are expressed as

41a $$\begin{aligned} {\bar{d}} _{ij}^{2}= & {} \frac{1}{{2{V_2}}}\int _S {\left( {{n_i}v_j^{2} + {n_j}v_i^{2}} \right) } dS = {D _m}{\delta _{ij}}, \end{aligned}$$

41b $$\begin{aligned} {\bar{d}} _{ij}^{1}= & {} \frac{1}{{2{V_1}}}\int _S {\left( {{n_i}v_j^{1} + {n_j}v_i^{1}} \right) } dS = \frac{{{D _m}\left( {3{\kappa _2} + 4{\mu _2}} \right) }}{{3{\kappa _1} + 2\left( {2 + {\kappa _{s}}} \right) {\mu _2}}}{\delta _{ij}}, \end{aligned}$$

where

42a-e $$\begin{aligned} \kappa _{s}^{}\mathrm{{ = }}\frac{{ {{\kappa _0}} }}{{a{\mu _2}}},\quad \mu _{s}^{}\mathrm{{ = }}\frac{{\mu _0^{}}}{{a {\mu _2 }}},\quad {\eta _s} = \frac{{{\eta _0}}}{{a^3\mu _2 }},\quad \kappa _0=2(\mu _0+\lambda _0), \quad {\eta _{0}} = {3{\zeta _s} + 5{\chi _s}}. \end{aligned}$$

Then the microscopic equivalent strain rate and stress of the micro RVE are written as

43a $$\begin{aligned} {\bar{d}} _{ij}^{3}= & {} \left( {1 - f} \right) {\bar{d}} _{ij}^{2} + f{\bar{d}} _{ij}^{1}, \end{aligned}$$

43b $$\begin{aligned} {\bar{\sigma }} _{ij}^{3}= & {} f\left( {{\bar{\sigma }} _{ij}^{1} + {{{\bar{\tau }} }_{ij}}} \right) + (1 - f){\bar{\sigma }} _{ij}^{2} = 3{\kappa _{3 }}{\bar{d}}_{ij}^{3}, \end{aligned}$$

where

44 $$\begin{aligned} {{{\bar{\tau }} }_{ij}} = \frac{1}{{{V_1}}}{\int _s {\left[ \sigma \right] } _{ki}}{n_k}{x_j}dA. \end{aligned}$$

Combining equations ( 43,44), the macroscopic equivalent bulk modulus can be obtained

45 $$\begin{aligned} {\kappa _{3}} = \frac{{ - 3{\kappa _1}\left( {3{\kappa _2} + 4f{\mu _2}} \right) - 2{\mu _2}\left( {{\kappa _2}\left( {6 - 6f + 3{\kappa _{s}}} \right) + 4f{\kappa _{s}}{\mu _2}} \right) }}{{9\left( { - 1 + f} \right) {\kappa _1} - 6\left( {2 + {\kappa _{s}}} \right) {\mu _2} + f\left( { - 9{\kappa _2} + 6{\kappa _{s}}{\mu _2}} \right) }}. \end{aligned}$$

Considering that the inclusion is a nanovoid, its bulk modulus ($\kappa _1$) and shear modulus ($\mu _1$) are both 0. Since the microscopic matrix is von Mises matrix, the microscopic bulk modulus is infinite. The macroscopic equivalent bulk modulus can be further simplified

46 $$\begin{aligned} {\kappa _{3}}=\frac{{2\left( {2 - 2f + {\kappa _s}} \right) {\mu _2}}}{{3f}}. \end{aligned}$$

### Macroscopic equivalent shear modulus ($\mu _3$)

The macroscopic shear modulus is only affected by the macroscopic deviatoric strain rate:

47 $$\begin{aligned} {\textbf{D}} = {D_e}{\mathbf{{e}}_x}{\mathbf{{e}}_x} + {D_e}{\mathbf{{e}}_y}{\mathbf{{e}}_y} - 2{D_e}{\mathbf{{e}}_z}{\mathbf{{e}}_z}. \end{aligned}$$

To this end, the microscopic velocity field subjected only to the macroscopic deviatoric strain rate field is expressed as

48a $$\begin{aligned} v_R^1= & {} \left( {{F_{11}}\frac{{{R^2}}}{{{a^2}}} + {F_{12}} + {F_{13}}\frac{{{a^3}}}{{{R^3}}} + {F_{14}}\frac{{{a^5}}}{{{R^5}}}} \right) R{P_2}\left( {\cos \varphi } \right) , \end{aligned}$$

48b $$\begin{aligned} v_\varphi ^1= & {} \left( {{G_{11}}\frac{{{R^2}}}{{{a^2}}} + {G_{12}} + {G_{13}}\frac{{{a^3}}}{{{R^3}}} + {G_{14}}\frac{{{a^5}}}{{{R^5}}}} \right) R{\partial _\varphi }{P_2}\left( {\cos \varphi } \right) , \end{aligned}$$

48c $$\begin{aligned} v_R^2= & {} \left( {{F_{21}}\frac{{{R^2}}}{{{a^2}}} + {F_{22}} + {F_{23}}\frac{{{a^3}}}{{{R^3}}} + {F_{24}}\frac{{{a^5}}}{{{R^5}}}} \right) R{P_2}\left( {\cos \varphi } \right) , \end{aligned}$$

48d $$\begin{aligned} v_\varphi ^2= & {} \left( {{G_{21}}\frac{{{R^2}}}{{{a^2}}} + {G_{22}} + {G_{23}}\frac{{{a^3}}}{{{R^3}}} + {G_{24}}\frac{{{a^5}}}{{{R^5}}}} \right) R{\partial _\varphi }{P_2}\left( {\cos \varphi } \right) , \end{aligned}$$

48e,f $$\begin{aligned} v_\theta ^1 = 0,\quad v_\theta ^2 = 0, \end{aligned}$$

where ${P_2}\left( {\cos \varphi } \right)$ is the Legendre polynomial of order two.

Taking into account the geometric and constitutive equations (24a,b, 25a,b), substitute the microscopic stress into the stress balance equation:

49a-b $$\begin{aligned} \sigma _{ij,i}^1=0,\quad \sigma _{ij,i}^2=0. \end{aligned}$$

Based on equation (49a-b), eight unknown coefficients in the microscopic velocity field can be determined as

50a,b $$\begin{aligned} {G_{11}} = \frac{{5{F_{11}}{\lambda _1} + 7{F_{11}}{\mu _1}}}{{6{\lambda _1}}},\quad G_{12} = \frac{{{F_{12}}}}{2}, \end{aligned}$$

50c,d $$\begin{aligned} {G_{13}} = \frac{{{F_{13}}{\mu _1}}}{{3{\lambda _1} + 5{\mu _1}}},\quad {G_{14}} = - \frac{{{F_{14}}}}{3}, \end{aligned}$$

50e,f $$\begin{aligned} {G_{21}} = \frac{{5{F_{21}}{\lambda _2} + 7{F_{21}}{\mu _2}}}{{6{\lambda _2}}},\quad {G_{22}} = \frac{{{F_{22}}}}{2}, \end{aligned}$$

50g,h $$\begin{aligned} {G_{23}} = \frac{{{F_{23}}{\mu _2}}}{{3{\lambda _2} + 5{\mu _2}}},\quad {G_{24}} = - \frac{{{F_{24}}}}{3}. \end{aligned}$$

There are still 8 unknown coefficients in the microscopic velocity field that need to be determined by boundary conditions. First, the kinematic equations need to be satisfied on the boundary of the microscopic RVE.

51 $$\begin{aligned} v_R^2\left| {_{R = b}} \right. = 2{D _e}b \end{aligned}$$

By calculation, the form of the two coefficients can be determined

52a,b $$\begin{aligned} {F_{21}} = 0,\quad {F_{22}} = 2{D _e}. \end{aligned}$$

Second, the microscopic velocity field at the center of the microscopic RVE should avoid singularity, which requires that

53a,b $$\begin{aligned} {F_{14}} = 0,\quad {F_{13}} = 0. \end{aligned}$$

Third, the microscopic velocity field at the interface should satisfy the continuity condition:

54a,b $$\begin{aligned} v_R^1\left| {_{R = a} = } \right. v_R^2\left| {_{R = a}} \right. ,\quad v_\varphi ^1\left| {_{R = a} = } \right. v_\varphi ^2\left| {_{R = a}}. \right. \end{aligned}$$

By solving equation (54a,b), two unknown coefficients can be obtained

55a $$\begin{aligned} {F_{12}}= & {} - \frac{{7\left( {{\lambda _1} + {\mu _1}} \right) }}{{5{\lambda _1}}}{F_{11}} + \frac{2}{5}\left( {1 + \frac{{3{\mu _2}}}{{3{\lambda _2} + 5{\mu _2}}}} \right) {F_{23}} + 2{D _e}, \end{aligned}$$

55b $$\begin{aligned} {F_{24}}= & {} - \frac{{\left( {2{\lambda _1} + 7{\mu _1}} \right) }}{{5{\lambda _1}}}{F_{11}} - \frac{{9\left( {{\lambda _2} + {\mu _2}} \right) }}{{5\left( {3{\lambda _2} + 5{\mu _2}} \right) }}{F_{23}}. \end{aligned}$$

Finally, the stress condition containing the Steigmann–Ogden surface model needs to be satisfied at the interface.

56 $$\begin{aligned} {\left[ \sigma \right] _{ij}}{n_i} = {\tau _{ij,i}} + {T_{lu}}{\left( {{M_{kl,k}}{n_j}} \right) _{,u}} - \frac{2}{a}{M_{ml,m}}{n_j}{n_l} \end{aligned}$$

According to solving equation (56), the specific expressions of $F_{11}$ and $F_{23}$ can be finally obtained.

Given the definition of the microscopic strain rate equation (40), the microscopic average strain rate of the matrix and inclusions can be written as

57a $$\begin{aligned} {\bar{d}}_{ij}^2= & {} - {D_e}{\mathbf{{e}}_x}{\mathbf{{e}}_x} - {D_e}{\mathbf{{e}}_y}{\mathbf{{e}}_y} + 2{D_e}{\mathbf{{e}}_z}{\mathbf{{e}}_z}, \end{aligned}$$

57b $$\begin{aligned} {\bar{d}}_{ij}^1= & {} - {D_b}{\mathbf{{e}}_x}{\mathbf{{e}}_x} - {D_b}{\mathbf{{e}}_y}{\mathbf{{e}}_y} + 2{D_b}{\mathbf{{e}}_z}{\mathbf{{e}}_z}, \end{aligned}$$

57c $$\begin{aligned} {D_b}= & {} \frac{{5{F_{12}}{\lambda _1} + 7{F_{11}}\left( {{\lambda _1} + {\mu _1}} \right) }}{{10{\lambda _1}}}. \end{aligned}$$

Based on the average microscopic strain rate, the average stress can be expressed as

58a $$\begin{aligned} {\bar{d}} _{ij}^{3}= & {} \left( {1 - f} \right) {\bar{d}} _{ij}^{2} + f{\bar{d}} _{ij}^{1}, \end{aligned}$$

58b $$\begin{aligned} {\bar{\sigma }} _{ij}^{3}= & {} f\left( {{\bar{\sigma }} _{ij}^{1} + {{{\bar{\tau }} }_{ij}}} \right) + (1 - f){\bar{\sigma }} _{ij}^{2} = 2{\mu _{3 }}{\bar{d}}_{ij}^{3}, \end{aligned}$$

In terms of equations (57, 58, 42a-e), the macroscopic equivalent shear modulus can be easily derived

59 $$\begin{aligned} \mu _3 = \frac{{2\left( {{l_3} + 2{l_4}} \right) - 3{f}\left( {{l_1} + 2{l_2}} \right) }}{{2\left( {{l_3} + 2{l_4}} \right) + 2{f}\left( {{l_1} + 2{l_2}} \right) }}\mu _2, \end{aligned}$$

where

60a,b $$\begin{aligned} {l_1} = \left( {2 + {\kappa _s}} \right) \left( {1 - {\mu _s}} \right) ,\quad {l_2} = {\eta _s}\left( {1 - {\kappa _s} - 3{\mu _s}} \right) , \end{aligned}$$

60c,d $$\begin{aligned} {l_3} = 3 + 2{\kappa _s} + 3{\mu _s} + {\kappa _s}{\mu _s},\quad {l_4} = {\eta _s}\left( {3 + {\kappa _s} + 3{\mu _s}} \right) . \end{aligned}$$

Finally, by substituting equations (59, 45) into equation (20), the macroscopic yield criterion of porous metals with Steigmann–Ogden surface model can be finally derived.

## Results and discussion

In the previous section, we derived the yield criterion of porous materials by using homogenization approach and Steigmann–Ogden surface model. The yield criterion is analytical and implicit with respect to the macroscopic mean and equivalent stresses. The purpose of this section is to explore the effect of the parameters of the Steigmann–Ogden surface model, the radius of the nanovoid and the porosity on the macroscopic yield criterion. For the microscopic RVE, the matrix material is treated as aluminum with the shear modulus $\mu _2=23.6$ GPa and the yield strength $\sigma _Y=250$ MPa. Following Tian’s research^31^, two sets of surface bulk modulus and shear modulus are considered for nanovoids surface: Case 1 ($\kappa _0=12.95$ nN/nm, $\mu _0=-0.376$ nN/nm) and Case 2 ($\kappa _0=-3.86$ nN/nm, $\mu _0=-5.43$ nN/nm). For the surface bending moduli of nanovoids, which are additionally considered in the Steigmann–Ogden surface model, three surface parameters are taken into account ($\eta _0=0,-30,-60$nN nm). For comparison, the classical solutions without considering the surface effects are also listed in the figure as much as possible.

**Figure 2:**
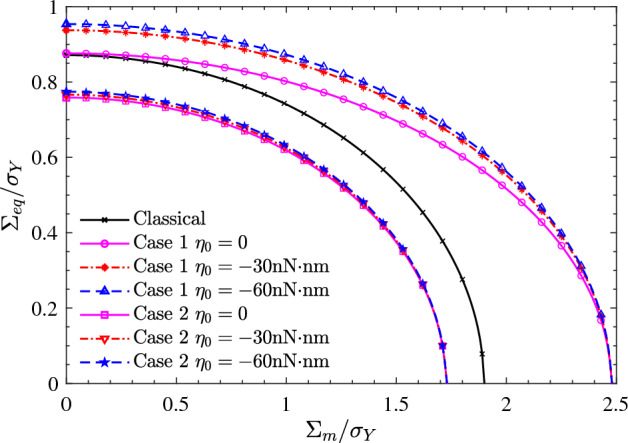
Effect of surface bending modulus ($\eta _0$) on the macroscopic yield loci of nanoporous aluminum. The porosity and the nanovoid radius are taken as $f = 0.1$ and $a=1$ nm. Two sets of surface bulk and shear moduli are considered: Case 1 ($\kappa _0=12.95$ nN/nm, $\mu _0=-0.376$ nN/nm) and Case 2 ($\kappa _0=-3.86$ nN/nm, $\mu _0=-5.43$ nN/nm).

Figure 2 shows the macroscopic yield loci of nanoporous aluminum at different surface bending moduli. The porosity and the nanovoid radius are taken as $f = 0.1$ and $a=1$ nm. It can be clearly observed that Case 1 effectively increases the mean and equivalent stresses of the macroscopic yield loci, while Case 2 reduces the macroscopic mean and equivalent stresses. When the surface bending modulus ($\eta _0$) is taken as 0, the Steigmann–Ogden surface model degenerates to the Gurtin-Murdoch surface model. It can therefore be concluded from Fig. 2 that the surface bending modulus only affects the equivalent stress of the macroscopic yield loci, not the mean stress. The equivalent stress of the macroscopic yield loci is amplified regardless of the change in the surface bending modulus.

**Figure 3:**
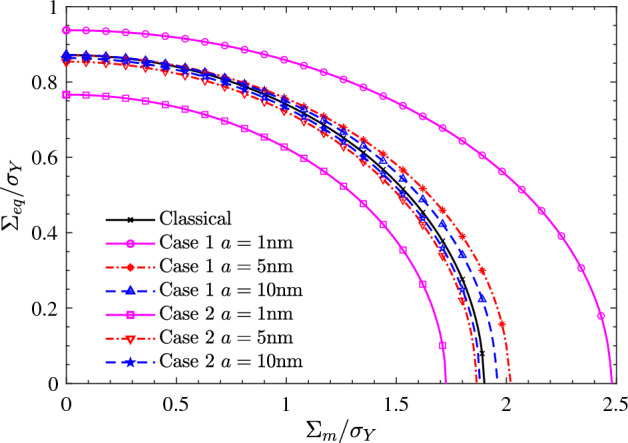
Effect of nanovoids radius (*a*) on the macroscopic yield loci of nanoporous aluminum. The porosity and the surface bending modulus are taken as $f = 0.1$ and $\eta _0=-30$nN $\cdot$ nm. Two sets of surface bulk and shear moduli are considered: Case 1 ($\kappa _0=12.95$ nN/nm, $\mu _0=-0.376$ nN/nm) and Case 2 ($\kappa _0=-3.86$ nN/nm, $\mu _0=-5.43$ nN/nm).

Figure 3 depicts the macroscopic yield loci of nanoporous aluminum at different nanovoid radii. The porosity and the surface bending moduli are taken as $f = 0.1$ and $\eta _0=-30$nN nm, respectively. A phenomenon worth noting is that regardless of the value of the nanovoid radius, Case 1 enlarges the macroscopic yield loci of nanoporous aluminum, while case2 narrows the macroscopic yield loci. Regardless of Case 1 or Case 2, with the increase of the nanovoid radius, the surface effect will decay rapidly and approach the classical solution. Compared with Case 1, Case 2 can effectively change the mean stress of the macroscopic yield loci while the effect on the equivalent stress is very limited.

**Figure 4:**
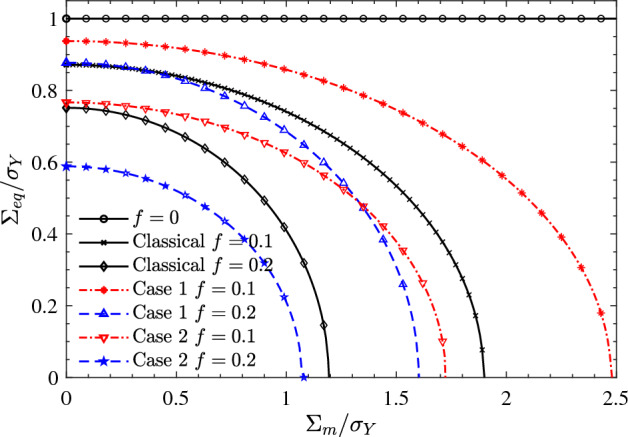
Effect of porosity (*f*) on the macroscopic yield loci of nanoporous aluminum. The nanovoids radius and the surface bending modulus are taken as $a = 1$nm and $\eta _0=-30$nN nm. Two sets of surface bulk and shear moduli are considered: Case 1 ($\kappa _0=12.95$ nN/nm, $\mu _0=-0.376$ nN/nm) and Case 2 ($\kappa _0=-3.86$ nN/nm, $\mu _0=-5.43$ nN/nm).

Figure 4 shows the macroscopic yield loci of nanoporous aluminum subjected to different porosity. The nanovoids radius and the surface bending modulus are taken as $a = 1$nm and $\eta _0=-30$nN nm, respectively. It can be observed that when the porosity increases, the macroscopic yield loci shrink significantly. When the porosity is taken to be 0, the surface effect does not exist. And the macroscopic yield loci of nanoporous aluminum will degenerate into the von Mises yield loci. Another obvious phenomenon is that Case 1 significantly increases the equivalent stress of the macroscopic yield loci, while Case 2 has a very weak effect on the equivalent stress. As the porosity increases, the amplifying effect of Case 1 on the equivalent stress of the macroscopic yield loci is continuously enhanced.

## Concluding remarks

In this paper, we developed a macroscopic yield criterion for nanoporous materials based on the homogenization approach and Steigmannn–Ogden surface model. The RVE is described as the classic Mori-Tanaka model, that is, an infinite matrix containing a tiny nanaovoid. The surface effects of nanovoids are applied at the interface between the nanovoid and the matrix. Firstly, based on the homogenization approach, the macroscopic yield criterion of nanoporous materials is obtained, which includes the dependence of the macroscopic equivalent modulus and the microscopic uniform modulus. Secondly, based on the establishment of the trial velocity field, the macroscopic equivalent modulus including the influence of the Steigmannn–Ogden model can be obtained. Finally, an implicit macroscopic yield criterion for nanoporous materials is derived. Based on the surface modulus, porosity and nanopore radius, related studies were developed and analyzed in detail. On the basis of extensive parametric studies, a few major conclusions can be drawn as follows.

- Different surface moduli will have different regulation effects on the macroscopic yield criterion of nanoporous materials. Positive surface moduli significantly increase the macroscopic yield loci, while negative surface moduli decrease the macroscopic yield loci slightly.
- The surface bending modulus only affects the equivalent stress of the macroscopic yield loci and has no effect on the mean stress.
- The influence of surface effects on the macroscopic yield criterion of nanoporous materials strongly depends on the size of the nanovoid radius. The smaller the radius of the nanopore, the more obvious the surface effect.
- The macroscopic yield criterion of nanoporous materials strongly depends on the size of the porosity. The larger the macroscopic porosity, the more obvious the shrinkage of the macroscopic yield loci.

## Data Availability

The datasets used and analysed during the current study available from the corresponding author on reasonable request.
